# Two Approaches for Evaluating the Effects of Galangin on the Activities and mRNA Expression of Seven CYP450

**DOI:** 10.3390/molecules24061171

**Published:** 2019-03-25

**Authors:** Yin-Ling Ma, Feng Zhao, Jin-Tuo Yin, Cai-Juan Liang, Xiao-Li Niu, Zhi-Hong Qiu, Lan-Tong Zhang

**Affiliations:** 1 Department of Pharmaceutical Analysis, School of Pharmacy, Hebei Medical University, Shijiazhuang 050017, China; maling-shz@163.com (Y.-L.M.); zhaofeng-37@163.com (F.Z.); yinjintuo@163.com (J.-T.Y.); caijuanliang@126.com (C.-J.L.); 2 National Clinical Drug Monitoring Center, Department of Pharmacy, Hebei Province General Center, Shijiazhuang 050051, China; niuxl0327@163.com (X.-L.N.); 15930818866@126.com (Z.-H.Q.)

**Keywords:** CYP450 enzyme, cocktail probe drug, RT-PCR, LC-MS/MS, galangin

## Abstract

Galangin is a marker compound of honey and *Alpinia officinarum* Hance that exhibits great potential for anti-microbial, anti-diabetic, anti-obesity, anti-tumour and anti-inflammatory applications. Galangin is frequently consumed in combination with common clinical drugs. Here, we evaluated the effects of galangin on cytochrome P450 (CYP)-mediated metabolism, using two different approaches, to predict drug–drug interactions. Male Sprague Dawley rats were administered galangin daily for 8 weeks. A “cocktail-probes” approach was employed to evaluate the activities of different CYP450 enzymes. Blood samples of seven probe drugs were analysed using liquid chromatography-tandem mass spectrometry in positive and negative electrospray-ionisation modes. Pharmacokinetic parameters were calculated to identify statistical differences. CYP mRNA-expression levels were investigated in real-time quantitative polymerase chain reaction experiments. The galangin-treated group showed significantly decreased AUC_0–∞_ and C_max_ values for CYP1A2, and CYP2B3. The galangin-treated group showed significantly increased AUC_0–∞_ and C_max_ values for CYP2C13 and CYP3A1. No significant influences were observed in the pharmacokinetic profiles of CYP2C11, CYP2D4 and CYP2E1. The mRNA-expression results were consistent with the pharmacokinetic results. Thus, CYP450 enzyme activities may be altered by long-term galangin administration, suggesting galangin to be a promising candidate molecule for enhancing oral drug bioavailability and chemoprevention and reversing multidrug resistance.

## 1. Introduction

Cytochrome P450 (CYP450) is a phase I metabolic enzyme that is expressed in multiple biological organs. It is mainly responsible for transforming endogenous and exogenous substances, including drugs, in vivo. When the activity of cytochrome P450 is disturbed, it can affect the metabolic links of corresponding substrates and cause various biological effects [1]. By evaluating the activity of CYP450, we can predict possible drug–drug interactions, drug–food interactions and the compatibility mechanisms of Chinese herbs in vivo, and provide valuable clinical information for drug combinations and compatibility with the daily diet.

Galangin, a natural flavonoid, is a marker compound of honey and *Alpinia officina rum* Hance (Zingiberaceae family) [2], which exhibits great potential in terms of its anti-microbial [3], anti-diabetic [4], anti-obesity [5], anti-tumour [6], anti-inflammatory properties [7], anti-oxidant [8], anti-osteoporosis [9] and lipid regulating effects [10]. Based on the wide application of galangin in food, medicine and health care products, and the lack of research on the biological interactions of galangin, this study was designed to investigate the effects of galangin on the CYP1A2, CYP2B3, CYP2C11, CYP2C13, CYP2D4, CYP2E1 and CYP3A1 enzymes in rats. First, liquid chromatography/tandem mass spectrometry (LC-MS/MS) was used to establish a method for simultaneously probing the levels of seven drugs in rat plasma samples: phenacetin, bupropion, diclofenac acid, omeprazole, dextromethorphan, chlorzoxazone and midazolam. Then, probes were used to study the effects of galangin on the activities of seven metabolic enzymes. Finally, real-time fluorescence quantitative polymerase chain reaction (PCR)was used to evaluate galangin mRNA expression. The effects of galangin on the expression of seven metabolic enzymes in rats were comprehensively analysed, and potential interactions between galangin and drug combinations were predicted.

## 2. Results and Discussion

### 2.1. High-Performance Liquid Chromatography–Tandem Mass Spectrometry (HPLC-MS/MS) Method Development

Different HPLC-MS/MS methods for simultaneous quantitative determination of probe drugs have been published [11,12]. However, only four methods employed simultaneous quantitative assays with multiple probes [11,12,13,14]. Lu et al. [11] established an HPLC-MS/MS method for evaluating the activity of seven CYP isoenzymes (CYP1A2, 2B6, 2C9, 2C19, 2D6, 2E1 and 3A4) in rats, but negative-ionization mode is not sensitive enough for probing drugs such as phenacetin, bupropion and omeprazole. Kim et al. [12] used a gradient elution method to detect most metabolites in positive electrospray ionisation (ESI) mode. Li et al. [13] adopted two methods combining different HPLC systems, namely, one method that coupled a high-pressure chemical-ionization interface with MS, with the other method being negative ESI mode. Nevertheless, previous investigators [12,13] concluded that matrix effects were absent. The matrix effect is an important issue in LC-MS method development. The method by De Bock et al. [14] adopted the approach of monitoring and detecting CYP activity in either positive or negative ESI mode, which has a certain practical value. Nevertheless, two runs, one in positive-ionisation mode and one in negative-ionisation mode, were necessary in order to detect all metabolites.

**Figure 1:**
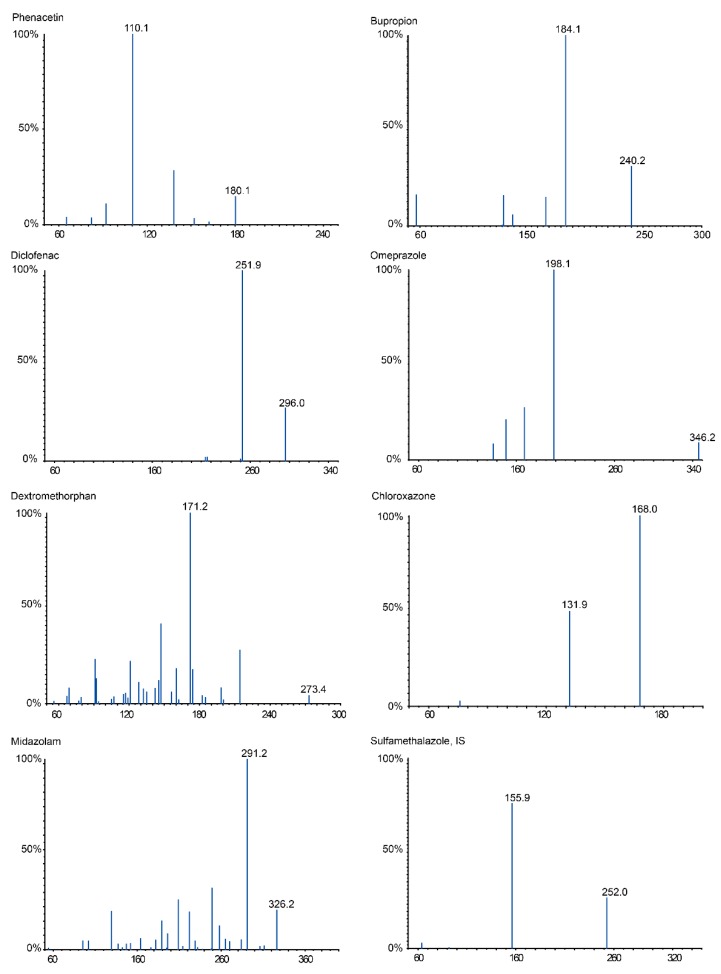
The MS/MS spectra of seven analytes and IS in positive and negative ion mode.

**Figure 2:**
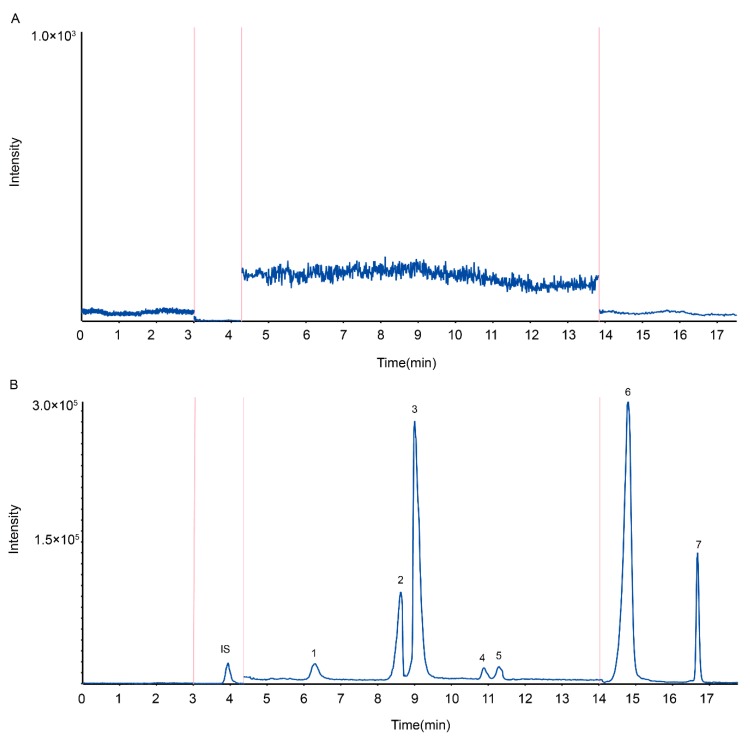
Chromatograms of the analytes and IS in positive ion mode. Note: (**A**) blank plasma; (**B**) sample plasma 1 h after administration of cocktail solution; (1) bupropion; (2) omeprazole; (3) phenacetin; (4) midazolam; (5) dextromethorphan; (6) chloroxazone; (7) diclofenac and IS.

Recently, we used an LC-MS method to quantify seven probe drugs. A more intense and stable signal for the seven probe drugs and the internal standard (IS) was observed by ESI, in positive and negative ion-switching mode. The precursor and daughter ions were selected and the MS/MS parameters were optimized to give the highest response in multiple-reaction monitoring (MRM) mode (Figure 1 and Table 1). A Wonda Cract ODS-2 C18 Column was employed to provide better performance for the peaks of the seven analytes in this study. A mobile phase consisting of water fortified with 0.1% formic acid enhanced the response and improved the peak shape. Considering the chemical diversity in the structures of the metabolites, a mobile phase with a gradient elution was employed to obtain better peak shapes and a shorter run time for the seven analytes and the IS, using water containing 0.1% formic acid as eluent B. Eluent A (methanol + 0.1% formic acid) was increased gradually from 45% to 90% during the course of 17.5 min, using a flow rate of 1.0 mL/min. A column temperature of 25 °C was selected to obtain a symmetric peak shape. A chromatogram in both ionization modes is shown in Figure 2. All peaks were baseline-separated.

### 2.2. Method Validation

A sensitive, rapid, simple and economical HPLC-MS/MS method was developed and validated for the simultaneous quantification of seven probe drugs. The method-validation procedure was based on the Guidance for Industry Bioanalytical Method Validation of the European Medicines Agency and the U.S. Food and Drug Administration (FDA) [15]. The probe drugs for phenacetin (CYP1A2), bupropion (CYP2B3), diclofenac (CYP2C11), omeprazole (CYP2C13), dextromethorphan (CYP2D4), chlorzoxazone (CYP2E1) and midazolam (CYP3A1) together with the IS sulfamethoxazole (STZ), were separated at 25 °C on a Wonda Cract ODS-2 C18 column (4.6 mm × 150 mm, inside diameter [i.d.], 5.0 μm). A gradient elution (total run time of 17.5 min) was performed, using methanol containing 0.1% (*v*/*v*) formic acid (A) and water containing 0.1% (*v*/*v*) formic acid (B) at a flow rate of 1.0 mL/min.

Calibration curves showed good linearity over the range of 1.006–2414.4 ng/mL for phenacetin (*r* = 0.9933), 0.801–403.2 ng/mL for bupropion (*r* = 0.9902), 1.01–808ng/mL for diclofenac (*r* = 0.9892), 1.1015–812 ng/mL for omeprazole (*r* = 0.9918), 0.99–247.5 ng/mL for dextromethorphan (*r* = 0.9952), 1.287–514.8 ng/mL for chlorzoxazone (*r* = 0.9904), and 2.005–6416 ng/mL for midazolam (*r* = 0.9967). The lower limits of detection of phenacetin, bupropion, diclofenac, omeprazole, dextromethorphan, chlorzoxazone, and midazolam were 1.006, 0.801, 1.01, 1.015, 0.99, 1.287 and 2.005 ng/mL, respectively. The concentrations of phenacetin, bupropion, diclofenac, omeprazole, dextromethorphan, chlorzoxazone, and midazolam in rat plasma were simultaneously determined using an HPLC-MS/MS method (Figure 2). As shown in Table 2, the intra-day and inter-day precision of the method were within 9.7%, and the accuracy ranged from 91.9% to 113.2%. The extraction recoveries for the analytes were greater than 81.4% (Table 3). All variations in the matrix effect were within the range of 84.3% to 111.1% (Table 3). The lower limit of quantification (LLOQ) was consistent with the intended application, and no relative matrix effects were observed. In addition, the sample extracts were stable under various storage conditions (Table 4). The feasibility of this method was demonstrated by calculating the pharmacokinetic parameters of the probe and CYP450 activities.

### 2.3. Selection of CYP450 Isozymes

The liver is the most important scavenging organ for drugs and exogenous substances. Drugs are mainly metabolized by CYP enzymes in the liver. In the human body, the main CYP enzymes involved in drug metabolism include CYP1A2, CYP2B6, CYP2C9, CYP2C19, CYP2D6, CYP3A4 and CYP2E1, among which CYP2C9, CYP2D6 and CYP3A4 account for approximately 50% of the total liver CYP enzyme levels and can metabolize nearly 80% of all clinical drugs [16]. Human CYP1A2, CYP2B6, CYP2C9, CYP2C19, CYP2D6, CYP3A4 and CYP2E1 share high homology with rat CYP1A2, CYP2B3, CYP2C11, CYP2C13, CYP2D4, CYP3A1 and CYP2E1, respectively [17]. CYP1A2 is mainly distributed in the liver, accounting for 13% of the total CYP450. CYP1A2 is the main metabolic enzyme of warfarin, theophylline, clozapine, haloperidol and other drugs with a narrow therapeutic window [18]. The activity of CYP1A2 may change the exposure level of the above drugs in vivo and cause serious adverse drug reactions. CYP2B3 participates in the metabolism of approximately 7% of clinical drugs in vivo, including the anti-cancer drugs cyclophosphamide and tamoxifen, the anti-HIV drug Faviron, the anti-depressant imipramine, the intravenous anaesthetics propofol and ketamine, and the analgesic pethidine. CYP2B3 is also involved in the metabolism of carcinogens and environmental toxicants [19], making it an important exogenous metabolic enzyme. CYP3A4 is one of the most abundant CYP450 isoenzymes in the human body. Macrolactone antibiotics, antifungal agents, 3-hydroxy-3-methyl-glutaryl-coenzyme A (HMG-CoA) inhibitors, benzodiazepines, proton pump inhibitors, calcium channel blockers and other common clinical drugs are metabolized through CYP3A4 [20]. The CYP2E1 isoenzyme is a potent source for oxidative stress. Oxidative stress is critical for the pathogenesis of diseases and CYP2E1 is a major contributor to oxidative stress. When taking the above-mentioned medications, it is possible to take Chinese herbal medicine or dietary supplements containing galangin or related ingredients at the same time to induce or inhibit metabolic enzymes, which may lead to fluctuations of the therapeutic effect or an increase in metabolite concentrations and subsequent adverse reactions. In this study, seven rat-related isoenzymes were selected: CYP1A2, CYP2B3, CYP2C11, CYP2C13, CYP2D4, CYP3A1, and CYP2E1. The activities of the seven main CYP450 enzymes were determined using a sensitive, accurate, and reliable probe method.

### 2.4. Effect of Galangin on the Activities of Rat Liver CYPs

**Figure 3:**
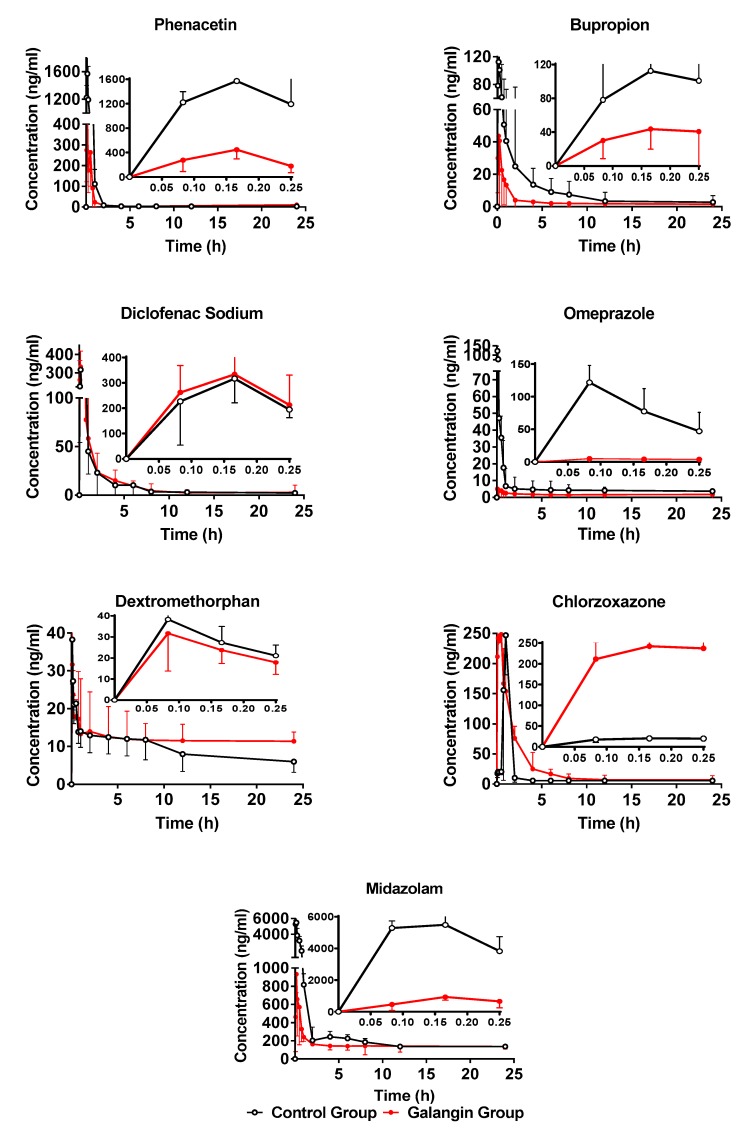
Mean plasma concentration–time curves of seven analytes in rats (mean ± *SD*, *n* = 6).

The plasma samples were collected and determined using the established method. The plasma concentration at each time point was calculated based on the standard curve. Average drug–time curves of the blank group and the drug-delivery group for the seven probe drugs were drawn using GraphPad prism 7.0.0 software (Figure 3). Pharmacokinetic parameters were calculated and analysed using DAS 3.2.4 and SPSS 21.0 software, respectively (Table 5). Compared with the control group, the CYP isoenzymes of the galangin group showed significant changes after 8 weeks of galangin administration.

#### 2.4.1. Effect of Galangin on Rat Hepatic CYP1A2

The pharmacokinetic profiles of phenacetin after long-term galangin treatments were used to describe the activity of CYP1A2. The pharmacokinetic parameters of phenacetin in the galangin-treatment groups in rats are shown in Table 5. The mean plasma concentration–time curves of phenacetin in two groups are presented in Figure 3. Compared with the control group, the AUC_0–t_ of phenacetin decreased significantly in the experimental group after 8 weeks of continuous gavage with galangin. Compared with the control group, the AUC_0–∞_, C_max_, and T_1/2_ values decreased by 72.33% (*p* < 0.01), 70% (*p* < 0.01) and 0.56-fold (*p* < 0.05), and CL_Z/_F increased by 5.27-fold (*p* < 0.01). Thus, galangin significantly induced the activity of CYP1A2. Therefore, when taking warfarin, theophylline, clozapine or haloperidol, attention should be paid to the combination with galangal or its components.

#### 2.4.2. Effect of Galangin on Rat Hepatic CYP2B3

Compared with the control group, the AUC_0–t_ of amphetazone decreased significantly in the experimental group after 8 weeks of continuous gavage of galangin. Compared with the control group, the AUC_0–∞_ and C_max_ values decreased by 67.86% (*p* < 0.01) and 42.89% (*p* < 0.01), respectively. The CL_Z_/F increased by 3.2-fold (*p* < 0.01). These results suggest that continuous administration of galangin can induce the CYP2B3 enzyme in the rat liver and accelerate drug metabolism.

#### 2.4.3. Effect of Galangin on Rat Hepatic CYP2C13

Compared with the control group, the AUC_0–∞_ value of omeprazole in the experimental group decreased significantly. Compared with the control group, the AUC_0–∞_ value increased 1.27-fold (*p* < 0.05), the C_max_ increased 1.66-fold (*p* < 0.05), and the T_1/2_ value decreased to 34.6% of the control group (*p* < 0.05) (Table 5). These results suggest that continuous administration of galangin can inhibit CYP2C13 enzyme activity in the rat liver, thereby slowing down drug metabolism.

#### 2.4.4. Effect of Galangin on Rat Hepatic CYP3A1

Compared with the control group, the AUC_0–t_ value of the drug–time curve of midazolam in the experimental group was significantly lower than that in the control group after 8 weeks of continuous gavage with galangin. Compared with the control group, the AUC_0–∞_ and C_max_ values decreased by 0.42-fold (*p* < 0.05) and 17.21% (*p* < 0.01), respectively, while the T_1/2_ and CL_Z_/F increased by 4.91-fold (*p* < 0.05) and 2-fold (*p* < 0.05), respectively. These results suggest that continuous administration of galangin can induce CYP3A1 enzyme activity in the rat liver and accelerate drug metabolism.

When taking drugs, such as macrolide antibiotics, antifungal agents, HMG-CoA reductase inhibitors, benzodiazepines, proton pump inhibitors or calcium channel blockers, it is possible to accelerate the metabolism of the corresponding medicines by consuming galangin or dietary supplements containing galangin or Chinese herbal medicines at the same time, which may lead to fluctuations in the therapeutic effect or increasing metabolite concentrations (causing adverse reactions), which should be paid close attention to.

#### 2.4.5. Effect of Galangin on Rat Hepatic CYP2C11, CYP2D4, and CYP2E1

Compared with the control group, the drug–time curves of diclofenac, dextromethorphan and chlorzoxazone in the experimental group were similar to those in the control group after 8 weeks of continuous gavage with galangin. Compared with the control group, the AUC_0–∞_, C_max_, T_max_, CL_Z_/F and T_1/2_ values were not significantly different (*p* > 0.05), indicating that galangin had no significant effect on the activities of CYP2C11, CYP2D4 and CYP2E1.

### 2.5. Effects of Galangin on Rat Liver CYP mRNA-Expression Levels

Flavonoids can activate the aromatic hydrocarbon receptor (AhR) [21], pregnane X receptor (PXR) [22] and constitutive androstane receptor (CAR) [23], thereby inducing CYP1A, CYP2B and CYP3A, and the corresponding CYP450 gene-expression level and protein-synthesis level are up-regulated accordingly, thus showing an inductive effect. Some flavonoids [24] showed strong cytotoxicity and inhibition, while some [24] had almost no effect on CYP gene-expression levels and enzyme activities. Therefore, it is speculated that galangin also induces expression of the CYP1A2, CYP2B1 and CYP3A1 genes by activating nuclear receptors. There are two main induction mechanisms of metabolic enzymes: the first is related to nuclear receptor-mediated transcription, and the second is related to mRNA or enzyme stability of mRNA after gene transcription. The mRNA expression of tumor necrosis factor-α (TNF-α)and transforming growth factor-β1(TGF-β1)were significantly increased in the fructose diet-fed rats, and galangin supplementation to fructose diet-fed rats downregulated the expression of these genes [8]. Apart from its antioxidant action, galangin has anti-inflammatory effects by affecting gene expression. This could be attributed to the fact that flavones and hydroxyflavones can inhibit the phosphorylation of proteins involved in the signal transduction [25].

**Figure 4:**
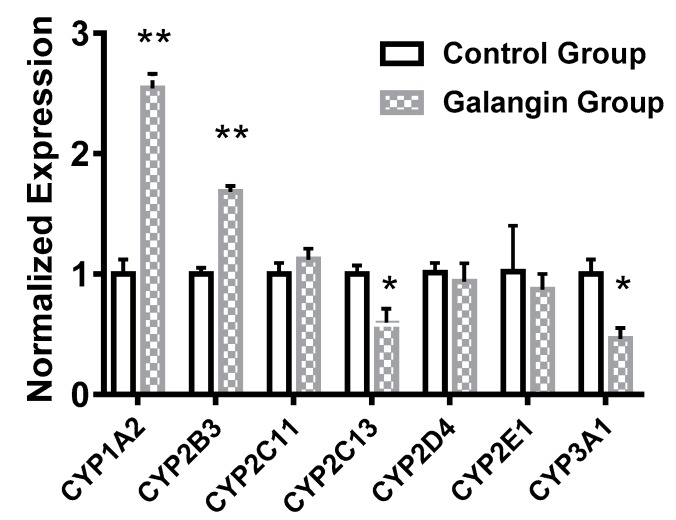
Effect of galangin on mRNA expression of CYP450 in rats (*n* = 4). * *p* < 0.05 versus control, ** *p* < 0.01 versus control.

Quantitative PCR was used to detect the effects of galangin on expression of the rat liver genes CYP1A2, CYP2B3, CYP2C11, CYP2C13, CYP2D4, CYP2E1 and CYP3A1 (Figure 4). Compared with the control group, the experimental group showed significantly increased expression of CYP1A2 and CYP2B3 gene (*p* < 0.01), which were up-regulated 2.54-fold and 1.68-fold in the experimental group, respectively. However, continuous administration of galangin did not significantly affect the expression of CYP2D4, CYP2C11 or CYP2E1 in rat livers (*p* > 0.05). Compared with the control group, the expression levels of CYP2C13 and CYP3A1 in the experimental group were down-regulated by 0.59-fold (*p* < 0.05) and 0.46-fold (*p* < 0.05), respectively.

These quantitative PCR results were consistent with those of the cocktail method.

## 3. Materials and Methods

### 3.1. Chemicals and Reagents

Galangin (98.3% purity) was obtained from Nanjing Plant Origin Biological Technology (Nanjing, China). Omeprazole (94.7% purity), chlorzoxazone (99.9% purity), and the IS sulfamethoxazole (99.6% purity) were purchased from National Institute for the Control of Pharmaceutical and Biological Products (Beijing, China). Dextromethorphan (98.4% purity) and bupropion (98.3% purity) were supplied by Dalian Meilun Biotechnology (Dalian, China). Phenacetin (98.5% purity) and diclofenac (98.5% purity) were purchased from Shanghai Macklin Biochemical Co., Ltd. (Shanghai, China). Midazolam (98.5% purity), and methanol and formic acid (LC-MS grade) were purchased from Sigma (St. Louis, MO, USA). Ultra-pure water was acquired from Wahaha Group Co., Ltd. (Hangzhou, China). Total RNA was extracted using the TRIzol reagent (Invitrogen, Carlsbad, CA, USA) and used for reverse transcription. Quantitative reverse transcription-polymerase chain reaction (PCR) analysis was performed with the ABI 7500 real-time PCR system (Applied Biosystems, Foster City, CA, USA). A Total RNA Kit was purchased from Tiangen Biotech Co., Ltd. (Beijing, China). PrimeScript^TM^ RT Master Mix was obtained from Takara Bio, Inc. (Kusatsu, Japan).

### 3.2. Animals and Experimental Design

Male Sprague Dawley rats (220–230 g, 8 weeks of age) were acquired from the Experimental Animals Center of Hebei Medical University (Shijiazhuang, China, animal certificate number: SCXK (Ji) 2018-003). The animal study was conducted based on the Guide for Care and Use of Laboratory Animals published by the National Institutes of Health (NIH publication no. 85–23, revised in 1985). Animals were maintained with ad libitum access to standard laboratory food (Diet composition: corn starch 60.0 g/100 g, casein (fat free) 20.0 g/100 g, methionine 0.7 g/100 g, groundnut oil 5.0 g/100 g, wheat bran 10.6 g/100 g, salt mixture 3.5 g/100 g, vitamin mixture 0.2 g/100 g) [8] and water in a breeding room with an ambient temperature 24 °C, a relative humidity of 60% and 12-h dark/light cycle (lights on from 08:00 to 20:00).

An 8 mg∙kg^−1^∙day^−1^ dose of galangin [26] was selected as an optimum dose to improve the antioxidant status and reduce hyperglycaemia in streptozotocin-induced diabetic rats. Twelve rats were assigned randomly to two groups of 6 animals each, namely the blank control group (CON) and the galangin-treated group (TRE). The CON group was treated with only 0.5% sodium carboxymethyl cellulose (CMC-Na) for 8 weeks. The TRE group was intragastrically treated 8 mg∙kg^−^^1^∙day^−^^1^ of galangin by using a ball-tipped incubation steel needle placed on a graded disposable syringe for 8 weeks in succession.

### 3.3. Pharmacokinetic Study

Twenty-four hours after the last administration of galangin, a cocktail solution, which contained phenacetin, diclofenac, dextromethorphan, midazolam (5 mg/kg), bupropion, omeprazole, and chlorzoxazone (10 mg/kg) in 0.5% CMC-Na solution, were administered orally to all rats in each group. Blood samples of each rat were collected from the posterior orbital veins at pre-dose (0 h), 0.08, 0.17, 0.25, 0.5, 0.75, 1, 2, 4, 6, 8, 12 and 24 h after oral administration. The blood samples (0.3 mL) were immediately transferred to heparinized tubes. Then 100 µL plasma were prepared from blood samples by centrifuging (4200 rpm, 15 min) and stored at −80°C until LC-MS/MS analysis.

### 3.4. Sample Preparation

Each 100 μL plasma sample was mixed with 20 μL of the IS working solution via vortexing for 1 min in a 1.5-mL centrifuge tube, after which 300 μL of methanol was added. Then, the resulting solution was extracted via vortexing for 3 min.

After centrifuging at 15,000 rpm at 4 °C for 10 min, the organic phase was transferred to another tube and evaporated to dryness under a gentle stream of N_2_ stream at 30 °C. The dried residue was reconstituted in 100 μL of methanol and vortexed for 1 min before being transferred to an autosampler vial for analysis. Drug and Statistics (DAS) software (version 3.2.4, Chinese Pharmacology Society, Shanghai, China) was employed to analyse the pharmacokinetics parameters.

### 3.5. Preparation of Calibration Curves and Quality Control (QC) Samples

Stock solutions of phenacetin, bupropion, diclofenac, omeprazole, dextromethorphan, chlorzoxazone, midazolam and the IS (each 100 μg/mL) were individually prepared in methanol.

The calibration standards solutions were serially diluted with methanol by blank plasma to concentrations of 1.006, 2.012, 10.06, 40.24, 402.4, 804.8, 1609.6 and 2414.4 ng/mL for phenacetin; 0.801, 2.034, 20.34, 101.7, 203.4 and 406.8 ng/mL for bupropion; 1.01, 2.02, 20.2, 202, 404 and 808 ng/mL for diclofenac; 1.015, 2.03, 10.15, 20.30, 203, 406 and 812 ng/mL for dextromethorphan; 1.287, 2.574, 12.87, 128.7, 257.4 and 514.8 ng/mL for chlorzoxazone; and 2.005, 4.01, 40.1, 401, 1604, 4010 and 6416 ng/mL for midazolam. The final concentration of the IS was 20 ng/mL.

QC samples were prepared by individually spiking blank rat plasma at three concentrations: low, medium or high (2.012, 201.2 or 1609.6 ng/mL for phenacetin; 2.304, 20.34 or 203.4 ng/mL for bupropion; 2.02, 20.2 or 202 ng/mL for diclofenac; 2.03, 20.3 or 406 ng/mL for omeprazole; 1.98, 9.9 or 99 ng/mL for dextromethorphan; 2.574, 12.87 or 257.4 ng/mL for chlorzoxazone; 4.01, 401 or 4010 ng/mL for midazolam). All solutions were prepared during the day prior to beginning the animal study and stored at 4 °C until analysis.

### 3.6. LC-MS Analytical Conditions

Samples were analysed on an Agilent 1200 series HPLC system (Agilent Technologies, Foster City, CA, USA), consisting of an autosampler, a degasser, a column compartment and a quaternary solvent delivery system. HPLC separations were carried out on a Wonda Cract ODS-2 C18 column (4.6 mm × 150 mm, i.d., 5.0 μm; SHIMADZU-GL, Kyoto City, Japan) at 25 °C. Linear gradient elution was performed using methanol containing 0.1% (*v*/*v*) (A) and water containing 0.1% (*v*/*v*) formic acid (B) as mobile phases, processed at a flow rate of 1.0 mL/min as follows: 0–11 min, 45% A; 11–12 min, 45%–90% A (linear); 12–17.5 min, 90% A; and then back to the initial A:B ratio of 45:55 (*v*/*v*). The injected volume for all samples was 10 μL.

For detection and quantification, MS detection was performed using an API 3200 Qtrap™ system (AB SCIEX, Foster City, CA, USA) equipped with Turbo V sources and a turbo ion spray interface. The ion spray voltages were set to 5500 V or −4500 V, the source temperature was maintained at 650 °C, the ion source gas (gas 1) pressure was 60 psi, the ion source gas (gas 2) pressure was 65 psi, the curtain gas (nitrogen) pressure was 30 psi, the collision cell-exit potentials were 5.0/−5.0 V, and the entrance potentials were 10.0/−10.0 V. The MS instrument was operated in MRM mode. Two ions were monitored for each molecule. The dwell time of each ion pair was held constant at 50 ms. The declustering potential and collision energy of the quantitative-optimization mode for the probe drugs and the IS sulfamethoxazole are described in Table 1. Analyst™ software (version 1.6.2 AB SCIEX, Foster City, CA, USA) was used for data acquisition and processing.

### 3.7. Method Validation

The method-validation procedure was based on the Guidance for Industry Bioanalytical Method Validation of the European Medicines Agency and the U.S. FDA [12]. Selectivity was determined by analysing twelve blank plasma samples in MRM to check for signals that might interfere with detection of the probe drug or the IS. In addition, two zero samples (blank samples including the IS) were analysed.

Twelve analyte-free plasma samples from different sources were analysed and checked for peaks interfering with the detection of the probe drug or the IS. The plasma samples did not contain any of the analytes.

Calibration curves were constructed at the different concentration ranges with a weighted (1/*x*^2^) least-squares linear regression, using the peak-area ratio (*y*) of each probe drug to that of the IS versus the concentrations (*x*). The percent deviation of the relative error (RE) from the nominal concentration (a measure of accuracy) and the relative standard deviation (RSD, a measure of precision) of the concentration defined as the LLOQ (considered as the lowest calibration standard) had to be <20%.

The intra- and inter-day precision and accuracy of the method were evaluated with QC samples and LLOQ samples at three different concentrations (six replicates each) on three consecutive days. The criteria for acceptable data included an accuracy within ±15% RE from the nominal values and a precision of within ±15% of the RSD. The RSD was acceptable if it was less than 20% of the LLOQ concentration.

The extraction recovery was investigated by comparing the mean peak areas of six samples spiked with low- and high-concentration QC samples for each probe drug before the extraction process with those obtained from samples spiked after the extraction.

The matrix effect was calculated by comparing analytes spiked into blank plasma extracts with the peak areas of the analytes in the mobile phase at an equivalent concentration.

The stability in plasma was evaluated by processing QC samples at three different concentrations in different conditions; long-term stability was evaluated after storage at −80°C for 15 days; short-term stability was evaluated after storage at room temperature for 2 h; freeze-thaw stability was evaluated after three freeze-thaw cycles from −80 °C to room temperature; post-preparative stability was evaluated by comparing QC samples analysed immediately after preparation and after 24 h at 4 °C or at room temperature for 4 h.

### 3.8. Effects of Galangin on mRNA Expression of CYP Enzymes in Rats

Galangin-treated and control animals were euthanised via decapitation at 48 h after the last administration (without fasting), after which livers were excised quickly, perfused with ice-cold 0.9% (*w*/*v*) sodium chloride to remove blood residue, weighted and stored at −80°C. Total RNA was extracted from rat liver samples with the Trizol Reagent (Invitrogen) in accordance with the manufacturer’s protocol. The RNA concentration was determined, and the quality of the isolated RNA was assessed based on the 260/280 nm absorbance ratio (1.8–2.0 indicates a highly pure sample). Subsequently, 5 µg of RNA for each sample was reverse transcribed to cDNA using a PrimeScript^TM^ RT Reagent Kit with gDNA Eraser (Kusatsu, Japan). The total RNA concentration of each reaction was 45 µg/mL. The reverse transcription conditions were as follows: gDNA was removed from the samples at 42 °C for 2 min, incubated at 4 °C for 10 min, reacted at 37 °C for 15 min, denatured at 85 °C for 5 s and held at 4 °C for 10 min. The obtained products were stored at −20°C. Reactions were performed in a final volume of 10 μL, according to the protocol recommended for the Power Up^TM^ SYBR^TM^ Green Master Mix Kit (Thermo Fisher Scientific, Vilnius, Lithuania). The amplification conditions were as follows: UDG enzyme activation at 50 °C for 2 min, and initial denaturation at 95 °C for 2 min followed by denaturation at 95 °C for 15 s, annealing at 60 °C for 15 s, extension at 72 °C for 1 min. Forty cycles were carried out. The relative mRNA-expression levels in the control and treated groups were calculated using the 2^−∆∆CT^ method. In this study, *GAPDH* was selected as the internal reference gene, and *CYP1A2*, *CYP2B3*, *CYP2C11*, *CYP2C13*, *CYP2D4*, *CYP2E1*, *CYP3A1* and *GAPDH* mRNA sequences were identified by searching National Center for Biotechnology Information (NCBI) NCBI’s Nucleotide database and some references. The sequences of the forward and reverse primers are shown in Table 6.

### 3.9. Statistical Processing Method

The data were analysed using SPSS software, version 21.0 (SPSS InC., Chicago, IL, USA). The pharmacokinetic parameters were calculated using DAS software, version 3.2.4 (version 3.2.4, Chinese Pharmacology Society, Shanghai, China). The average drug–time curves were drawn using GraphPad prism 7.0.0 software for Windows (GraphPad Software Inc., La Jolla, CA, USA). The parameters of the drug-treated groups were compared with those of the blank-control group using a t test and the non-parametric rank–sum test. A *p*-value < 0.05 was considered to reflect a statistically significant difference.

## 4. Conclusions

Enzyme induction and inhibition have significant effects on the drug treatment of galangin, especially the combination of drugs. When galangin is combined with drugs mainly metabolised by the CYP1A2 and CYP2B3 enzymes, the blood concentrations of these drugs may be reduced. When galangin is combined with drugs mainly metabolised by the CYP2C13 and CYP3A1 enzymes, the metabolism of these drugs will be slowed down, the active time will be prolonged, and the pharmacological activity or toxic side effects will be enhanced. The results of this study may provide more reliable experimental data and scientific explanations for the rational clinical application of related herbal (dietary supplement)–drug interactions.

## Figures and Tables

**Table 1:** MRM parameters for probe drugs and internal standards.

Enzyme Isoform	Probe Drug	Retention Time (min)	C (Probe Drug μmol/L)	MRM Condition			
				Precursor-Ion (*m*/*z*)	Daughter-Ion (*m*/*z*)	Fragment Energy (V)	Collision Energy (eV)
CYP1A2	Phenacetin	9.01	5	180.1	110.1	57.56	27.71
CYP2B3	Bupropion	6.29	10	240.2	184.1	64.64	17.15
CYP2C11	Diclofenac	16.68	5	296.0	251.9	−24.50	−16.50
CYP2C13	Omeprazole	11.31	10	346.2	198.1	31.38	15.29
CYP2D4	Dextromethor-phan	10.87	5	273.4	172.1	77.95	50.72
CYP2E1	Chloroxazone	14.79	10	168.0	131.9	−65.05	−27.00
CYP3A1	Midazolam	8.63	5	326.2	291.2	71.86	36.50
Internal standard	Sulfamethalaz-ole	3.99	-	252.0	155.9	−30.00	−20.00

**Table 2:** Intra- and inter-day precision and accuracy values for probe drugs in rat plasma at LLOQ, low, medium and high concentrations (*n* = 6).

Compounds	Concentration (ng/mL)	Precision RSD (%)		Accuracy (%)	
		Intra-Day	Inter-Day	Intra-Day	Inter-Day
Phenacetin	1.006	8.21	7.06	4.12	6.23
	2.012	5.96	4.51	−2.34	−4.04
	201.20	7.35	6.30	8.41	11.79
	1609.6	4.79	4.66	3.23	5.34
Bupropion	0.801	2.91	6.43	3.52	9.23
	2.034	7.34	5.25	6.14	9.35
	20.34	6.16	2.71	5.65	7.11
	203.4	2.44	3.55	−2.47	−3.76
Diclofenac	1.01	2.80	8.34	4.98	5.92
	2.02	1.21	5.26	6.90	9.45
	20.20	4.37	4.91	7.04	8.61
	202.00	3.55	4.27	4.93	5.85
Omeprazole	1.015	2.89	3.55	11.71	12.13
	2.03	4.10	2.78	7.26	6.41
	20.30	3.62	4.40	5.85	8.28
	406	1.23	6.05	2.92	8.66
Dextromethorphan	0.99	6.41	7.03	−4.16	−6.84
	1.98	5.12	6.31	6.97	12.02
	9.9	4.75	2.26	8.77	9.52
	99	3.69	1.85	8.55	10.61
Chloroxazone	1.287	5.81	4.53	6.83	7.13
	2.574	2.33	3.75	6.32	9.06
	12.87	2.69	5.97	2.91	4.75
	257.4	5.13	4.32	5.45	8.27
Midazolam	2.005	2.01	7.08	5.86	7.85
	4.01	1.89	4.93	−3.7.83	−8.33
	401	1.13	6.12	3.39	4.27
	4010	0.71	0.63	4.11	8.31

**Table 3:** The mean extraction recoveries and matrix effect of the seven analytes and IS in rats plasma at low and high concentration (*n* = 6).

Compounds	Spiked Conc. (ng/mL)	Extraction Recovery (%)		Matrix Effect (%)	
		Mean ± SD	RSD (%)	Intra-Day	Inter-Day
Phenacetin	2.01	81.48 ± 5.97	7.33	102.33 ± 7.45	7.22
	1609.60	90.01 ± 3.26	3.62	92.25 ± 4.27	4.64
Bupropion	2.03	88.95 ± 6.84	7.61	93.51 ± 6.30	6.56
	203.40	94.92 ± 3.40	3.22	111.13 ± 4.16	3.77
Diclofenac	2.02	92.74 ± 4.25	3.67	85.63 ± 4.65	4.84
	404	90.20 ± 4.58	8.98	89.02 ± 8.03	3.99
Omeprazole	2.03	91.66 ± 5.25	4.51	85.64 ± 10.13	6.89
	406	93.49 ± 5.75	8.76	91.21 ± 4.63	4.56
Dextromethorphan	1.98	93.54 ± 3.68	12.58	95.23 ± 54.0	5.34
	99	94.61 ± 4.33	10.31	97.04 ± 6.21	6.30
Chloroxazone	2.574	93.52 ± 5.46	3.82	88.01 ± 7.82	5.46
	257.4	101.24 ± 5.17	9.82	86.84 ± 6.74	6.47
Midazolam	4.01	93.33 ± 5.06	4.63	84.34 ± 4.96	2.66
	4010.00	92.43 ± 2.36	6.36	89.16 ± 4.03	4.40

**Table 4:** Stability of seven probe drugs in rat plasma under various storage conditions (*n* = 3).

Compounds	Spiked conc. (ng/mL)	Blood Sample Stored at RT for 2 h		Blood Sample Stored at		Blood Sample for Freeze-Thawing 3 Cycles		Post−Preparative Sample Stored at 4 °C for 24 h		Post−Preparative Sample Stored at RT for 4 h	
				−80 °C for 15 Days							
		Calc. conc	Accuracy	Calc. conc	Accuracy	Calc. conc	Accuracy	Calc. conc	Accuracy	Calc. conc	Accuracy
		(ng/mL)	(%)	(ng/mL)	(%)	(ng/mL)	(%)	(ng/mL)	(%)	(ng/mL)	(%)
Phenacetin	10.08	9.79 ± 0.20	−2.9	9.85 ± 1.51	−2.25	9.73 ± 0.24	−3.5	9.97 ± 0.28	0.28	9.77 ± 0.50	−2.26
	201.2	203.6 ± 6.66	1.22	205.00 ± 12.53	1.89	192.30 ± 5.20	−4.42	196.96 ± 5.13	−2.12	195.72 ± 17.41	−2.73
	1609.6	1630.19 ± 61.37	1.28	1560.30 ± 12.44	−3.06	1564.93 ± 32.09	−2.78	1576.26 ± 38.13	−2.07	1561.74 ± 35.53	−2.97
Bupropion	2.3	2.42 ± 0.28	4.91	2.38 ± 0.23	3.33	2.22 ± 0.39	−3.33	2.35 ± 0.26	2.17	2.25 ± 0.35	−2.32
	20.34	20.47 ± 1.92	0.62	20.55 ± 1.92	1.02	20.98 ± 2.57	3.13	20.54 ± 0.18	0.97	19.93 ± 1.45	−2.02
	203.4	198.67 ± 12.84	−2.32	212.26 ± 22.29	4.36	196.30 ± 17.49	−3.49	206.79 ± 30.07	1.67	201.45 ± 29.70	−0.96
Diclofenac	2.02	1.95 ± 0.19	−3.63	1.99 ± 0.43	−1.65	2.05 ± 0.27	1.32	2.07 ± 0.12	2.64	2.06 ± 0.20	2.15
	20.2	19.75 ± 2.76	−2.22	20.52 ± 1.06	1.6	20.58 ± 1.42	1.88	20.45 ± 3.53	1.25	19.75 ± 2.76	−2.22
	404	401.17 ± 19.48	−0.7	404.99 ± 50.97	0.24	409.20 ± 29.18	1.29	406.63 ± 39.90	0.65	395.24 ± 14.96	−2.17
Omeprazole	2.03	2.09 ± 0.31	2.79	2.05 ± 0.34	0.99	2.00 ± 0.33	−1.48	2.07 ± 0.10	1.81	1.99 ± 0.24	−1.97
	203	205.96 ± 35.04	1.46	202.63 ± 30.88	−0.18	209.30 ± 29.66	3.1	202.63 ± 30.88	−0.18	200.91 ± 35.05	−1.02
	406	392.14 ± 32.82	−3.41	397.14 ± 35.26	−2.18	400.47 ± 38.99	−1.36	403.81 ± 23.84	−0.54	401.22 ± 31.33	−1.52
Dextromethorp−han	1.98	1.96 ± 0.16	−1.04	1.99 ± 0.12	0.67	1.93 ± 0.13	−2.53	2.01 ± 0.14	1.51	1.95 ± 0.33	−1.52
	9.9	9.88 ± 0.22	−0.24	10.03 ± 0.23	1.31	9.99 ± 2.02	0.88	9.76 ± 0.45	−1.45	9.65 ± 0.22	−2.49
	99	99.92 ± 2.14	0.93	97.2 ± 2.56	−1.82	97.87 ± 5.15	−1.14	97.55 ± 4.77	−1.99	100.38 ± 12.69	1.39
Chloroxazone	2.574	2.57 ± 0.37	−1.18	2.56 ± 0.13	0.43	2.53 ± 0.20	−0.54	2.59 ± 0.22	1.83	2.45 ± 0.31	−3.93
	12.87	12.99 ± 0.44	−1.63	13.12 ± 0.71	1.97	12.79 ± 0.44	−0.6	12.79 ± 1.24	−0.6	12.53 ± 1.43	−2.67
	257.4	250.20 ± 2.88	0.96	252.73 ± 17.27	−1.82	251.16 ± 10.37	−2.42	254.5 ± 16.71	−1.13	253.83 ± 17.43	−1.39
Midazolam	4.01	3.98 ± 0.29	−0.75	3.96 ± 0.27	−1.33	3.94 ± 0.35	−1.75	399.03 ± 11.57	2.16	4.08 ± 0.17	1.75
	401	395.70 ± 22.26	−1.32	405.7 ± 26.34	1.17	405.70 ± 25.59	1.17	399.03 ± 11.57	−0.49	406.67 ± 15.79	1.41
	4010	3969.06 ± 233.65	−1.02	4062.30 ± 108.90	1.3	4095.64 ± 87.80	2.14	3962.30 ± 66.21	−1.18	3995.64 ± 112.78	−0.36

**Table 5:** Pharmacokinetic parameters of seven analytes in rat plasma after a single oral administration of a probe drugs solution in rats (*n* = 6).

Analytes	Group	AUC_(0–t)_ (μg·h/L)	AUC_(0–∞)_ (μg·h/L)	C_max_(ug/L)	T_max_(h)	T_1/2_ (h)
Phenacetin	Blank	1194.97 ±620.95	1276.56 ±617.86	1442.54 ±250.87	0.14 ±0.04	2.46 ±0.94
	Treat	327.63 ** ±228.18	353.22 ** ±224.12	433.42 ** ±147.95	0.16 ±0.06	1.38 * ±1.45
Bupropion	Blank	165.25 ±2.94	201.94 ±24.20	101.5 ±14.49	0.22 ±0.04	3.25 ±1.18
	Treat	51.13 ** ±14.68	64.91 ** ±17.01	57.97 ** ±18.09	0.19 ±0.05	4.05 ±1.42
Diclofenac	Blank	386.52 ±132.62	400.40 ±127.67	425.78 ±124.74	0.12 ±0.04	1.38 ±0.46
	Treat	397.96 ±18.43	430.27 ±238.07	354.53 ±84.72	0.15 ±0.03	2.01 ±0.630
Omeprazole	Blank	75.30 ±12.22	129.70 ±23.10	95.76 ±35.93	0.083 ±0.01	10.86 ±4.08
	Treat	126.86 * ±29.32	165.33 * ±29.54	159.49 * ±58.26	0.081 ±0.01	3.76 * ±1.72
Dextrometho-rphan	Blank	298.32 ±8.06	463.11 ±88.17	32.76 ±19.27	0.14 ±0.04	22.57 ±8.07
	Treat	399 ±46.39	758.12 ±599.98	61.47 ±62.32	0.32 ±0.38	23.39 ±7.91
Chloroxazone	Blank	218 ±274.51	306.99 ±399.66	380.06 ±499.13.	0.50 ±0.52	2.38 ±0.78
	Treat	302 ±124.51	496.20 ±192.58	232.33 ±100.08	0.23 ±0.21	2.84 ±3.19
Midazolam	Blank	6454.1 ±1345.7	11194.82 ±6581.06	5252.44 ±654.62	0.11 ±0.04	3.61 ±1.62
	Treat	1558.15 * ±732.44	4712.50 * ±1748.06	903.98 ** ±255.58	0.15 ±0.03	17.74 ** ±6.22

Values are expressed as mean ± SD, *n* = 6. AUC_(0–∞)_—area under concentration-time curve extrapolated to infinity, T_1/2_—elimination half-time, T_max_—time to maximum concentration, C_max_—maximum concentration. * *p* < 0.05 vs. control. * *p* < 0.05 = significant difference in comparison to the control group (*t*-test). ** *p* < 0.01 = significant difference in comparison to the control group (*t*-test).

**Table 6:** Sequences of primers for RT-PCR analyses.

CYPs	Forward Primer Sequence	Reverse Primer Sequence
1A2	GTCACCTCAGGGAATGCTGTG	GTTGACAATCTTCTCCTGAGG
2B3	AGGACCCCGTCCCTTACC	CCGGCCAGAGAAAGCCTC
2C11	CTGCTGCTGCTGAAACACG	TTTCATGCAGGGGCTCCG
2C13	TGGTCCACGAGGTTCAGAGATACA	GGTTGGGAAACTCCTTGCTGTCAT
2D4	TGCGAGAGGCACTGGTGA	CGTGGTCCAAAGCCCGAC
2E1	GACCTTTCCCTCTTCCCATCCTTG	GTAGCACCTCCTTGACAGCCTTG
3A1	GGCAAACCTGTCCCTGTGAAAGA	CTGGCGTGAGGAATGGAAAGAGT
GAPDH	TGCTGAGTATGTCGTGGAG	GTCTTCTGAGTGGCAGTGAT
